# Real-world outcomes of subcutaneous infliximab in a Middle Eastern inflammatory bowel disease cohort: a prospective study of switch and de novo treatment strategies

**DOI:** 10.1093/crocol/otag067

**Published:** 2026-06-27

**Authors:** Maryam A Alahmad, Nadeen Mamon Omar, Mohammed Nabil Quraishi

**Affiliations:** Department of Gastroenterology, Sheikh Shakhbout Medical City, Abu Dhabi, United Arab Emirates; Department of Gastroenterology, Sheikh Shakhbout Medical City, Abu Dhabi, United Arab Emirates; Department of Gastroenterology, Sheikh Shakhbout Medical City, Abu Dhabi, United Arab Emirates; Institute of Cancer and Genomic Sciences, University of Birmingham, Birmingham, United Kingdom; College of Medicine and Health Sciences, Khalifa University of Science and Technology, Abu Dhabi, United Arab Emirates

**Keywords:** inflammatory bowel disease, Crohn’s disease, ulcerative colitis, infliximab, subcutaneous

## Abstract

**Background:**

Subcutaneous (SC) infliximab offers pharmacokinetic and convenience advantages over intravenous (IV) therapy, but real-world data from the Middle East are lacking. This study evaluated SC infliximab outcomes in a prospective middle eastern inflammatory bowel disease (IBD) cohort.

**Methods:**

A prospective observational cohort study was conducted in the United Arab Emirates. Adult IBD patients were enrolled as switchers (transitioning from stable IV to SC infliximab 120 or 240 mg Q2W) or new starters (SC 120 mg Q2W following IV induction). Clinical outcomes and pharmacokinetics were assessed.

**Results:**

Fifty-eight patients were included (33 switchers [10 receiving 120 mg Q2W, 23 receiving 240 mg Q2W] and 25 new starters), with median follow-up of 10.1 months (interquartile range [IQR] 7.3-12.9). In the switcher cohort, 90.9% maintained clinical remission post-switch, with stable C-reactive protein (CRP), serum albumin, and fecal calprotectin, and significant increases in median trough concentrations: 9.5-20.0 mcg/mL in the 120 mg group (*P* = .044) and 8.0-42.0 mcg/mL in the 240 mg group (*P* = .001). Among new starters, 88% achieved clinical remission post-induction, with a median post-induction trough level of 16.0 mcg/mL; significant improvements in CRP, serum albumin, and fecal calprotectin were observed. Dose intensification was required in 28% of new starters. Drug persistence was high and comparable across all groups (log-rank *P* = .61), with 12-month rates of over 92% across all cohorts.

**Conclusions:**

SC infliximab is effective for both switching and de novo IBD management in a middle eastern population, delivering high clinical remission rates, robust drug exposure, and durable treatment persistence.

## Graphical abstract

**Graphical Abstract:**
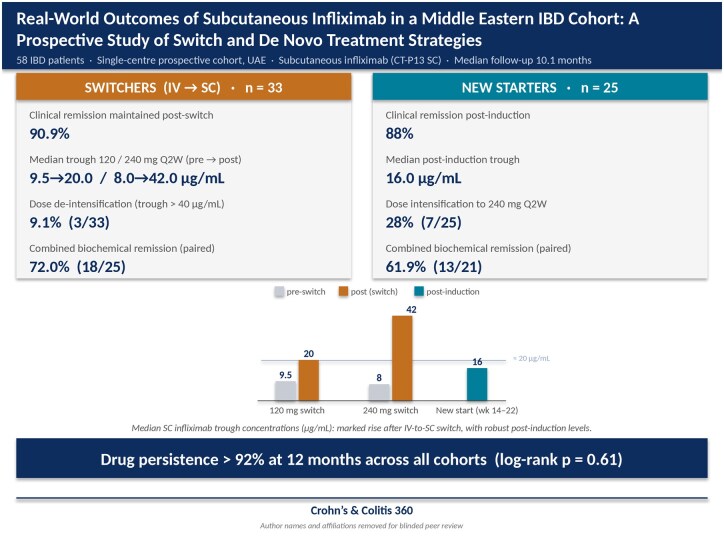

## Introduction

Intravenous (IV) infliximab has been a cornerstone of inflammatory bowel disease (IBD) therapy for over 2 decades, fundamentally transforming its management by improving rates of clinical remission and mucosal healing. Seminal trials such as ACCENT I and ACT 1 established its efficacy for both induction and maintenance of remission in Crohn’s disease (CD) and ulcerative colitis (UC) respectively.^1^^,^^2^ However, hospital-based IV infusions present logistical challenges for patients and healthcare systems, including time away from work, travel, and significant utilization of infusion center resources. Furthermore, the pharmacokinetic profile of IV administration is characterized by high peak and low trough concentrations, which has been associated with immunogenicity and subsequent loss of response.

The development of a subcutaneous (SC) formulation of the infliximab biosimilar CT-P13 was designed to address these limitations. At-home self-administration significantly enhances patient convenience and autonomy while reducing the burden on healthcare facilities. From a pharmacokinetic standpoint, SC infliximab provides more stable serum concentrations with consistently higher trough levels compared to its IV counterpart. Pivotal randomized controlled trials, including the CT-P13 SC 1.6 and LIBERTY studies, have established the pharmacokinetic non-inferiority and clinical efficacy of the SC formulation for maintaining remission in IBD.^3^^,^^4^ This improved pharmacokinetic profile is hypothesized to reduce immunogenicity and potentially lead to better long-term outcomes.

As clinical experience with SC infliximab has grown, a wealth of real-world evidence has emerged to validate the findings from controlled trials. Large observational cohort studies, such as the REMSWITCH study and others, have consistently confirmed that switching stable patients from IV maintenance to SC infliximab is safe, effective, and well-accepted.^5–7^ A key and consistent finding across these real-world cohorts is a significant increase in infliximab trough levels post-switch, even in patients who were on intensified IV regimens.^7^^,^^8^ This provides reassurance that standard SC dosing is adequate for the majority of patients.

Despite this growing body of evidence, significant knowledge gaps remain. The majority of published data, from both clinical trials and real-world settings, has focused on patients switching from established IV maintenance therapy. There is considerably less evidence on outcomes for patients newly initiated on SC infliximab following a standard IV induction regimen. Crucially, the pivotal SC infliximab studies and major real-world cohorts have predominantly enrolled patients from North American and European centers. There remains a significant lack of data on the use, effectiveness, and safety of SC infliximab in other populations, including patients from the Middle East.

This study evaluated the real-world clinical, pharmacokinetic, and safety outcomes of SC infliximab in a prospective IBD cohort from the Middle East, encompassing both patients switched from stable IV maintenance therapy and those newly initiated following IV induction.

## Materials and methods

### Study design and setting

This was a single-center, prospective, observational cohort study conducted at Sheikh Shakhbout Medical City (SSMC), Abu Dhabi, a tertiary IBD referral center in the United Arab Emirates. Data were extracted from the SSMC IBD Registry, a prospectively maintained electronic database capturing longitudinal clinical, pharmacokinetic, and biochemical data for all patients receiving biologic therapy. The study was conducted in accordance with the Declaration of Helsinki and received approval from the SSMC Institutional Review Board (SSMC-REC-585).

### Patient selection

Adult patients (18 years or older) with confirmed IBD (CD or UC) who initiated SC infliximab (CT-P13 SC; Remsima SC, Celltrion) between October 2024 and October 2025 were eligible for inclusion, provided a minimum of 3 months of follow-up was available. Patients with incomplete baseline data or early loss to follow-up were excluded.

Patients were enrolled into one of 2 cohorts:

- Switcher Cohort (*n* = 33): Patients electively switched from IV infliximab to SC infliximab, stratified by SC dose: 120 mg Q2W (*n* = 10) or 240 mg Q2W (*n* = 23).
- New Starter Cohort (*n* = 25): Patients with active IBD initiating infliximab, who received a 2-dose IV induction regimen (5 mg/kg at weeks 0 and 2) before transitioning to SC infliximab 120 mg Q2W maintenance from week 6.

Switching from IV to SC infliximab was offered only to patients in sustained clinical remission (Harvey–Bradshaw Index [HBI] <5 for CD or partial Mayo score [PMS] ≤2 for UC) and biochemical remission (C-reactive protein [CRP] <5 mg/L and/or fecal calprotectin <250 µg/g) maintained for at least 6 months. Pre-switch therapeutic drug monitoring was reviewed to confirm adequate drug exposure, although no fixed trough threshold gated the switch. Endoscopic remission was not mandatory but had been documented in the majority of patients as part of routine treat-to-target care.

### SC infliximab dose selection

Dose selection in the switcher cohort was guided by the REMSWITCH Algorithm (7), with final dose determined by shared clinical decision-making informed by therapeutic drug monitoring and disease phenotype. Patients on a standard IV regimen (5 mg/kg every 8 weeks) were switched to 120 mg Q2W. Those who had required dose intensification (10 mg/kg at any interval, shortened dosing intervals to every 4 or 6 weeks at 10 mg/kg, or doses exceeding 5 mg/kg at standard intervals) were transitioned to 240 mg Q2W. Patients on 5 mg/kg every 6 weeks were classified as borderline intensification and switched to 120 mg Q2W. All new starters received 120 mg Q2W as SC maintenance.

Where individual decisions departed from this default rule (a patient on 5 mg/kg Q8W escalated to 240 mg Q2W for active perianal disease with a low pre-switch trough; a patient on 10 mg/kg Q8W de-escalated to 120 mg Q2W for a supratherapeutic pre-switch trough), the rationale was documented and is reported. Dose escalation in the new starter cohort was therapeutic-drug-monitoring-driven, based on the post-induction infliximab trough measured at approximately week 14-22. Escalation from 120 to 240 mg Q2W was considered where this trough was below 20 µg/mL together with persistent clinical or biochemical disease activity. De-intensification from 240 to 120 mg Q2W was considered where the post-switch trough exceeded 40 µg/mL together with sustained clinical and biochemical remission.

### Data collection

Data extracted from the SSMC IBD Registry included: demographics (age, sex, body mass index [BMI]); disease characteristics (IBD subtype, Montreal classification, disease duration, and perianal involvement); treatment history (prior biologic exposure, and concomitant immunomodulator use); serum infliximab trough levels; clinical disease activity scores (HBI for CD; PMS for UC); and biochemical markers (CRP, serum albumin, and fecal calprotectin). These were recorded at baseline and at post-treatment reassessment, performed between week 12 and week 26 in new starters and at approximately 8-14 weeks after the switch in switchers; patients then continued routine clinical follow-up (median 10.1 months on SC infliximab overall). Serum infliximab trough levels were measured at steady state by validated ELISA as part of routine therapeutic drug monitoring. For switchers, pre-switch levels were obtained from the final IV infusion and post-switch levels at approximately 8-14 weeks after SC initiation. For new starters, post-induction levels were measured at approximately week 14-22.

### Primary outcomes

Clinical remission was defined as HBI below 5 (CD) or PMS of 2 or less (UC). Clinical response was defined as a reduction of 3 or more points in HBI or 2 or more points in PMS from baseline.

### Secondary outcomes

CRP normalization was defined as serum CRP below 5 mg/L, and calprotectin response as fecal calprotectin below 250 mcg/g. Combined biochemical remission required simultaneous achievement of both in patients with paired data. Drug persistence was defined as time from SC infliximab initiation to permanent discontinuation or switch back to IV for any reason; patients still on SC infliximab at last review were censored. Pharmacokinetic outcomes, perianal disease outcomes, dose modification rates, and the association between BMI and SC infliximab trough levels were also assessed as secondary endpoints.

Perianal disease outcomes were assessed by clinical examination, supported by pelvic MRI where this had been performed. Healing was defined as cessation of drainage from all perianal fistula tracts on gentle digital compression with no actively draining fistulae, sustained at the assessment timepoint. Improvement was defined as a reduction in the number of actively draining fistulae or a clear decrease in drainage and symptoms not meeting the definition of healing. Stable disease was defined as no change in fistula activity, and deterioration as a new perianal fistula or abscess, increased drainage, or a requirement for surgical or examination-under-anesthesia intervention.

### Subgroup analyses

Pre-specified subgroup analyses included: (1) the impact of BMI on SC infliximab trough levels (stratified as normal: below 25 kg/m^2^; overweight: 25-30 kg/m^2^; obese: 30 kg/m^2^ or above); (2) perianal CD outcomes across both cohorts; and (3) dose modification rates by cohort and dose group.

### Statistical analysis

Analyses were performed using R (R Core Team, Vienna, Austria). Continuous variables are reported as median (interquartile range [IQR]) and categorical variables as *n* (%). Paired comparisons used the Wilcoxon signed-rank test; between-group comparisons used the Mann–Whitney *U* test, Kruskal–Wallis test, or Fisher’s exact test as appropriate. BMI correlation with trough levels was assessed using Pearson’s correlation coefficient. Drug persistence was analyzed by the Kaplan–Meier method with between-group comparisons by log-rank test. All tests were 2-sided with significance set at *P* below .05. Non-missing denominators were used throughout, and missing data proportions are reported where applicable.

## Results

### Study population and baseline characteristics

A total of 58 patients were included: 33 IV-to-SC infliximab switchers (10 receiving 120 mg Q2W and 23 receiving 240 mg Q2W) and 25 new starters. Baseline characteristics are summarized in Table 1. The median age of the cohort was 30.4 (22.7, 36.7) years, and 57% of patients were male. Median BMI was 25.0 (22.0, 28.0) kg/m^2^. CD was the predominant diagnosis in 45/58 patients (78%), with UC present in 13/58 (22%). Among CD patients (*n* = 45), ileo-colonic disease (L3) was the most common location (67%), followed by ileal (L1: 20%) and colonic (L2: 13%) involvement, with upper GI modifier (L4) present in 11%. CD behavior was inflammatory (B1) in 64%, stricturing (B2) in 22%, and penetrating (B3) in 13%. Perianal disease was present in 23 of 45 CD patients (51%). Among UC patients (*n* = 13), left-sided colitis (E2) was present in 54% and pancolitis (E3) in 46% (*P* = .062 between groups). A significant between-group difference in disease duration was also observed (*P* = .010). Prior advanced therapy exposure was observed in 40% of the cohort overall, with no significant difference between groups (*P* = .13). Concomitant immunomodulator therapy was used in 52% of patients overall, with a significant difference between groups: new starters 80%, 120 mg Q2W switchers 20%, 240 mg Q2W switchers 35% (*P* < .001). Median pre-switch serum infliximab trough level (switcher cohort) was 8.0 (3.0, 29.0) mcg/mL. Median follow-up from initiation of SC infliximab was 10.1 months (IQR 7.3-12.9) across the entire cohort, with follow-up in the switcher cohort of 13.5 months (IQR 11.6-15.3) in the 120 mg Q2W group and 9.5 months (IQR 6.8-11.8) in the 240 mg Q2W group, compared to 8.9 months (IQR 7.3-12.9) in new starters (*P* = .011).

**Table 1:** Baseline characteristics of SC infliximab cohorts.

Characteristic	Overall *N* = 58	Switch to 120 mg Q2W (*N* = 10)	Switch to 240 mg Q2W (*N* = 23)	New starter (*N* = 25)	*P*-value
**Demographics**					
**Age at SC initiation (years)**	30.4 (22.7, 36.7)	29.7 (26.6, 39.9)	34.7 (26.7, 37.1)	24.3 (20.9, 33.6)	.13
**Sex**					>.9
** Female**	25 (43%)	5 (50%)	10 (43%)	10 (40%)	
** Male**	33 (57%)	5 (50%)	13 (57%)	15 (60%)	
**BMI (kg/m²)**	25.0 (22.0, 28.0)	23.0 (22.0, 25.0)	27.0 (23.0, 31.0)	25.0 (21.0, 28.0)	.12
**Disease characteristics**					
**Disease type**					.8
** CD**	45 (78%)	8 (80%)	19 (83%)	18 (72%)	
** UC**	13 (22%)	2 (20%)	4 (17%)	7 (28%)	
**CD location (Montreal classification)** ¹					.4
** Colonic (L2)**	6 (13%)	1 (13%)	4 (21%)	1 (5.6%)	
** Ileal (L1)**	9 (20%)	3 (38%)	2 (11%)	4 (22%)	
** Ileo-colonic (L3)**	30 (67%)	4 (50%)	13 (68%)	13 (72%)	
**Upper GI involvement (L4)** ^a^	5 (11%)	2 (25%)	2 (11%)	1 (5.6%)	.4
**CD behavior (Montreal classification)^a^**					.2
** Inflammatory (B1)**	29 (64%)	8 (100%)	10 (53%)	11 (61%)	
** Stricturing (B2)**	10 (22%)	0 (0%)	5 (26%)	5 (28%)	
** Penetrating (B3)**	6 (13%)	0 (0%)	4 (21%)	2 (11%)	
**Perianal disease** ^a^	23 (51%)	4 (50%)	9 (47%)	10 (56%)	>.9
**UC extent (Montreal classification)** ^b^					.062
** Left-sided colitis (E2)**	7 (54%)	0 (0%)	4 (100%)	3 (43%)	
** Pancolitis (E3)**	6 (46%)	2 (100%)	0 (0%)	4 (57%)	
**Disease duration (years)**	3.0 (1.3, 9.0)	4.1 (1.7, 9.1)	4.9 (2.1, 13.2)	1.6 (0.3, 6.7)	.010
**Treatment history**					
**Duration on IV infliximab (years)** ^c^	2.1 (1.3, 5.2)	2.0 (1.2, 5.3)	2.2 (1.3, 5.2)	—	.7
**Prior IV infliximab dose regimen** ^c^					<.001
** 5 mg/kg Q8W**	9 (27%)	8 (80%)	1 (4.3%)	—	
** 10 mg/kg Q4W**	11 (33%)	0 (0%)	11 (48%)	—	
** 10 mg/kg Q6W**	3 (9.1%)	0 (0%)	3 (13%)	—	
** 10 mg/kg Q8W**	9 (27%)	1 (10%)	8 (35%)	—	
** 5 mg/kg Q6W**	1 (3.0%)	1 (10%)	0 (0%)	—	
**Prior advanced therapies (pre-IFX)**					.13
** AT-naive**	35 (60%)	7 (70%)	17 (74%)	11 (44%)	
** 1 prior AT**	16 (28%)	3 (30%)	5 (22%)	8 (32%)	
** ≥2 prior AT**	7 (12%)	0 (0%)	1 (4.3%)	6 (24%)	
**Concomitant immunomodulator**	30 (52%)	2 (20%)	8 (35%)	20 (80%)	<.001
**Pharmacokinetics and follow-up**					
**Follow-up duration (months)**	10.1 (7.3, 12.9)	13.5 (11.6, 15.3)	9.5 (6.8, 11.8)	8.9 (7.3, 12.9)	.011
**Pre-switch infliximab level (μg/mL)** ^c^	8.0 (3.0, 29.0)	9.5 (5.8, 12.2)	8.0 (4.0, 32.0)	–	.7
**Post-switch/induction infliximab level (μg/mL)** ^d^	25.0 (16.0, 42.0)	20.0 (16.0, 33.0)	42.0 (26.0, 50.0)	16.0 (12.0, 27.0)	<.001

Values are median (IQR) for continuous variables and *n* (%) for categorical variables. *P-*value: Kruskal–Wallis test for continuous variables; Fisher’s exact test for categorical variables.

Abbreviations: AT, advanced therapy; CD, Crohn’s disease; IFX, infliximab; IQR, interquartile range; IV, intravenous; Q2W, every 2 weeks; Q4W, every 4 weeks; Q6W, every 6 weeks; Q8W, every 8 weeks; SC, subcutaneous; UC, ulcerative colitis.

a CD patients only: *N* = 45 overall (*N* = 8, 19, and 18 per subgroup, respectively). Percentages calculated among patients with available phenotype data.

b UC patients only: *N* = 13 overall (*N* = 2, 4, and 7 per subgroup, respectively).

c Switchers only (IV-to-SC group): *N* = 33 overall (*N* = 10 and 23 per subgroup). —, not applicable for new starters.

d Post-switch (switcher cohort) and post-induction (new starter cohort) trough concentrations reflect different pharmacokinetic phases and are not directly comparable.

### New starter cohort: Clinical and biochemical outcomes

**Figure 1:**
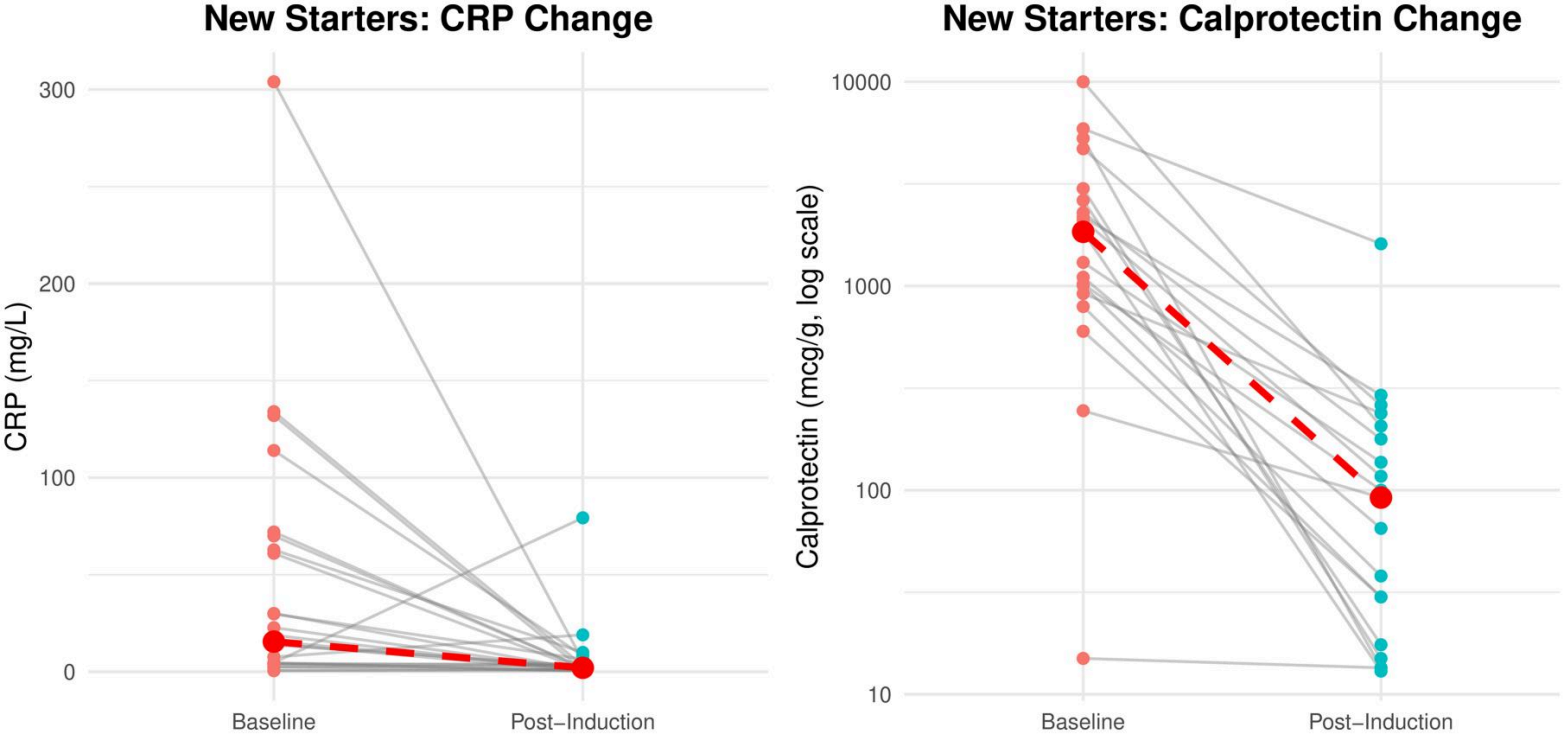
Biomarker response in new-start cohort. Changes in serum C-reactive protein (CRP, mg/L) and fecal calprotectin (FC, µg/g) from baseline to post induction in the new-start cohort. Individual patient trajectories are shown alongside group medians.

Following SC infliximab maintenance initiation (post-induction assessment measured between week 12 and week 26), 22/25 (88%) achieved clinical remission and 22/25 (88%) clinical response. Clinical disease activity scores improved significantly from baseline to post-induction assessment, with median scores decreasing from 9 (6, 12) to 1 (0, 2) (*P* < .001) (Table S1). Significant improvements in all biochemical markers of inflammation were observed following SC infliximab induction in the new starter cohort (Figure 1). Median serum CRP decreased from 15.5 (4.4, 62.8) mg/L at baseline to 2.0 (1.0, 4.0) mg/L post-induction (*P* = .001), with rates of CRP normalization (CRP below 5 mg/L) reaching 76%. Serum albumin improved from a median of 31.5 (29.0, 36.2) g/L to 41.0 (37.0, 44.0) g/L (*P* < .001). Fecal calprotectin decreased from a median of 1974.5 (983.2, 3000.0) mcg/g to 92.0 (30.0, 206.0) mcg/g (*P* < .001), with calprotectin response rates (below 250 mcg/g) increasing from 10% to 81%. Combined biochemical remission (simultaneous CRP normalization and calprotectin response) was achieved in 13/21 (61.9%) new starters.

### Switcher cohort: Clinical and biochemical outcomes

**Figure 2:**
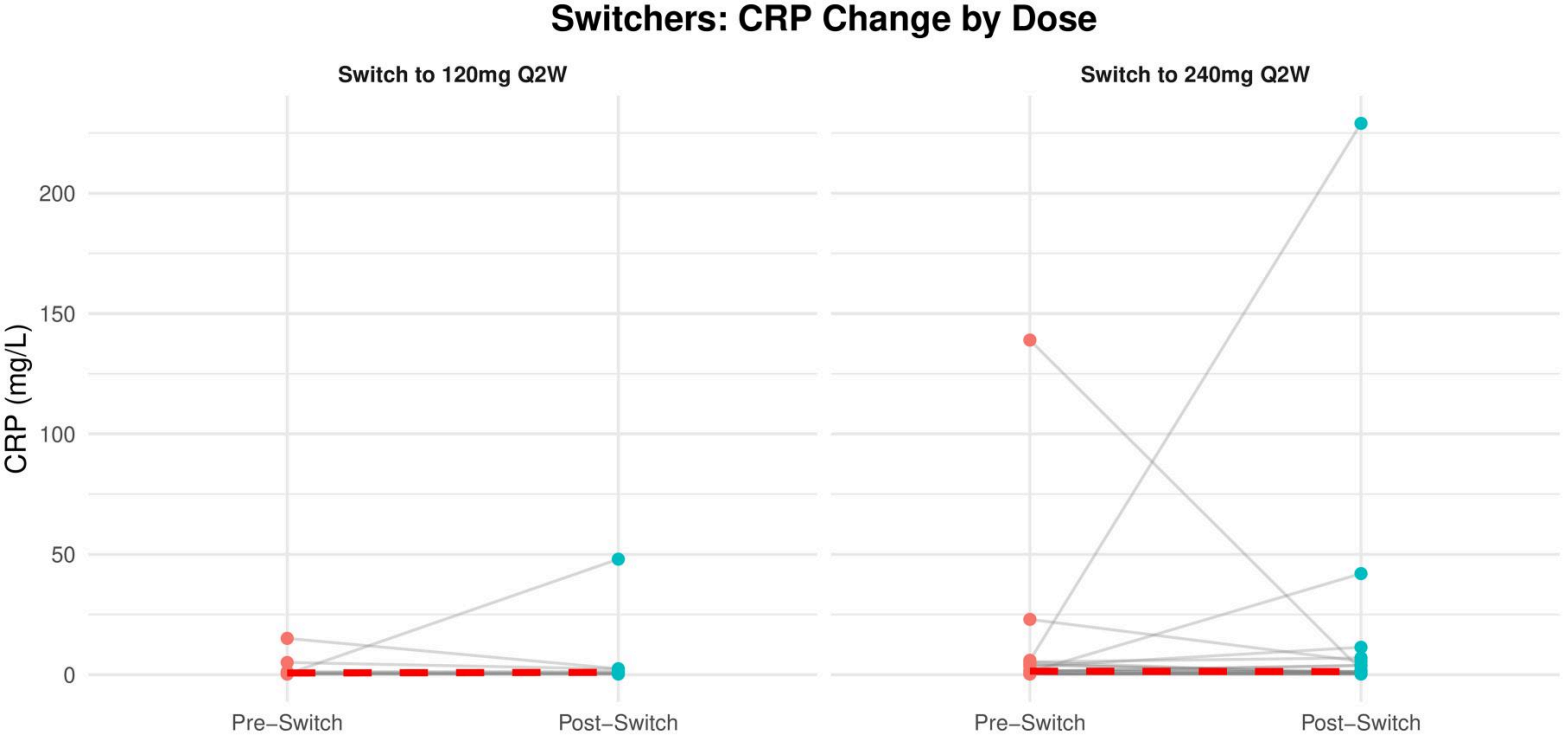
Biomarker response in the switcher cohort. Paired pre- and post-switch values for serum C-reactive protein (mg/L) and fecal calprotectin (µg/g) in the switcher cohort. Lines connect individual patients’ pre-switch and post-switch measurements.

All 33 switchers (100%) were in clinical remission at the time of transitioning from IV to SC infliximab, consistent with the study inclusion criterion. Following the switch, 30/33 (90.9%) maintained clinical remission at the post-switch assessment. Maintenance of remission was comparable between dose groups: 9/10 (90.0%) in the 120 mg Q2W group and 21/23 (91.3%) in the 240 mg Q2W group. Clinical disease activity scores remained stable from baseline to post-switch assessment (*P* = .326). Biochemical markers remained stable or showed modest improvements following the switch from IV to SC infliximab in the switcher cohort (Figure 2). Median serum CRP was 1.4 (0.3, 4.2) mg/L at baseline (pre-switch) and 1.3 (0.5, 3.8) mg/L post-switch (*P* = .954). Serum albumin increased from 40.0 (36.5, 42.5) g/L to 42.0 (39.0, 44.0) g/L (*P* = .018). Fecal calprotectin decreased from 81.0 (15.0, 454.0) mcg/g to 32.0 (28.0, 117.0) mcg/g (*P* = .023), with calprotectin response rates increasing from 72% to 84%. Combined biochemical remission was achieved in 18/25 (72.0%) switchers with paired data available for both markers.

### Perianal disease outcomes

Perianal disease was present in 23 CD patients across both cohorts. In the new starter cohort, 10 patients had perianal CD. Following SC infliximab initiation, perianal healing was achieved in 3/10 (30%), improvement was observed in 5/10 (50%), and perianal disease remained stable in 2/10 (20%), over a median follow-up of 9.7 months (IQR 5.2-14.9) in this subgroup. In the switcher cohort, 13 patients had perianal CD. Of these, 6/13 (46.2%) had already achieved perianal healing prior to switching (documented at baseline). Following the switch, an additional 4/13 (30.8%) achieved perianal healing, and 3/13 (23.1%) maintained stable perianal disease. No patients in the switcher cohort experienced perianal deterioration following the switch. Among switchers with CD (*n* = 27), SC infliximab trough levels were comparable between patients with perianal disease (median 37.5 [24.5, 45.0] mcg/mL) and those without perianal disease (median 33.0 [20.0, 47.0] mcg/mL). Maintenance of clinical remission was achieved in all 13/13 (100%) switchers with perianal disease, compared to 12/14 (85.7%) of those without perianal disease. Among new starters with perianal CD, post-induction trough concentrations were numerically higher in those achieving perianal healing (median 31.0 µg/mL) than in those whose disease improved or remained stable (median 15.5 µg/mL). Among switchers with perianal CD, post-switch troughs were uniformly high across all outcome categories with no deterioration, consistent with the comparable perianal and non-perianal troughs noted above.^9^ Given the small perianal CD subgroup, these estimates should be interpreted with caution.

### Pharmacokinetic outcomes

**Figure 3:**
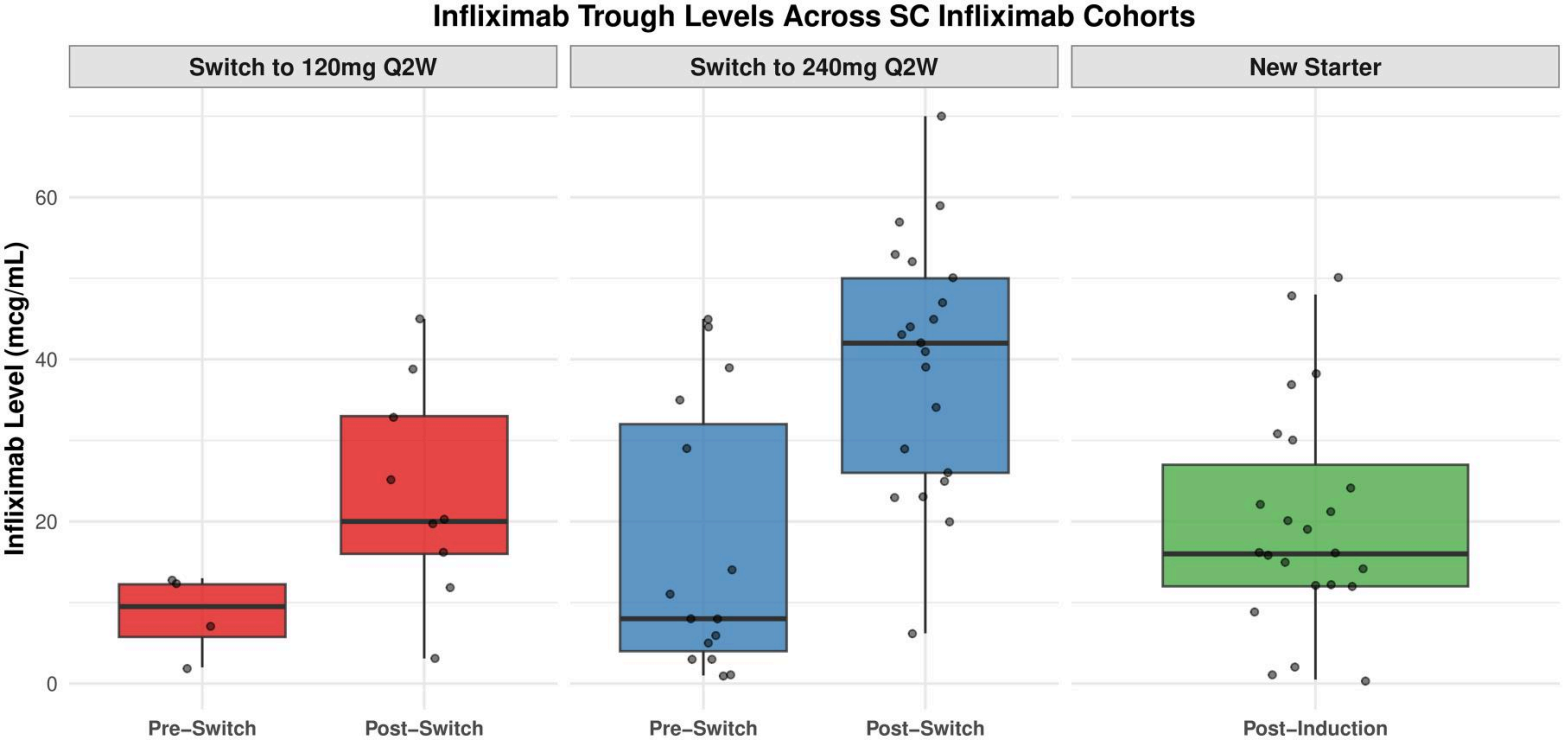
Infliximab trough levels for switcher and new-start cohorts. Boxplots illustrating the distribution of infliximab trough levels (µg/mL) for 3 patient groups. Pre-switch median levels were 9.5 µg/mL (120 mg group) and 8.0 µg/mL (240 mg group); post-switch medians were 20.0 and 42.0 µg/mL, respectively. New starters achieved a post-induction median of 16.0 µg/mL. Boxplots illustrating the distribution of infliximab trough levels (µg/mL) for 3 distinct patient groups. Switch to 120 mg Q2W (red): Patients who transitioned from maintenance intravenous (IV) infliximab to 120 mg subcutaneous (SC) infliximab every 2 weeks. Levels are shown before (“pre-switch”) and after (“post-switch”) the transition. Switch to 240 mg Q2W (blue): Patients who transitioned from maintenance IV infliximab to 240 mg SC infliximab every 2 weeks. Levels are also shown “pre-switch” and “post-switch.” New starter (green): Patients newly initiated on SC infliximab following a standard 2-dose IV induction. Levels are shown at week 14 (“post-induction”). The central line in each box represents the median value; the box itself spans the interquartile range (IQR), from the 25th to the 75th percentile. The whiskers extend to the most extreme data point which is no more than 1.5 times the IQR from the box. Individual points represent the trough level for each patient in the respective group.

Serum SC infliximab trough levels following the switch (switcher cohort) or post-induction (new starter cohort) are presented in Figure 3 and Table 1. In the switcher cohort, both SC dose regimens resulted in significantly higher trough levels compared to the preceding IV infliximab trough levels. For 120 mg Q2W switchers, median trough levels increased from 9.5 (5.8, 12.2) mcg/mL pre-switch to 20.0 (16.0, 33.0) mcg/mL post-switch (*P* = .044). For 240 mg Q2W switchers, median trough levels increased from 8.0 (4.0, 32.0) mcg/mL to 42.0 (26.0, 50.0) mcg/mL (*P* = .001). A significant difference in post-switch trough levels was observed between the 120 mg Q2W and 240 mg Q2W groups (*P* = .012). New starters achieved a median post-induction SC infliximab trough level of 16.0 (12.0, 27.0) mcg/mL. Post-induction (new starter) and maintenance-phase (switcher) trough concentrations reflect different pharmacokinetic phases and are not directly comparable; any apparent difference is descriptive and hypothesis-generating only. Among switchers who discontinued due to treatment failure, 3 cases were characterized by ongoing disease activity despite supratherapeutic trough levels exceeding 20 mcg/mL with no serological evidence of immunogenicity, consistent with pharmacodynamic rather than pharmacokinetic failure. Two patients developed neutralizing anti-infliximab antibodies despite combination therapy with azathioprine; both ultimately discontinued SC infliximab (see Drug Persistence section). The first had documented poor adherence, which likely contributed to immunogenicity. The second was a new starter with a prior history of IV infliximab exposure and discontinuation; on re-exposure during SC maintenance neutralizing antibodies were identified, indicating that antibody development likely preceded initiation of the SC formulation.

### Dose modifications

In the new starter cohort (*n* = 25), dose intensification (from 120 to 240 mg Q2W) was required in 7/25 (28.0%) patients. In the switcher cohort (*n* = 33), dose intensification (from 120 to 240 mg Q2W or higher frequency) was required in 2/33 (6.1%) patients due to low infliximab levels without evidence of loss of response, and dose de-intensification (from 240 to 120 mg Q2W) was performed in 3/33 (9.1%) patients on the basis of a supratherapeutic post-switch trough concentration above 40 µg/mL (observed troughs at de-intensification: 53, 57, and 70 µg/mL). Following de-intensification, these patients have been followed for a median of 12.1 months on SC infliximab (range 7.3-12.6 months) and have remained in clinical remission.

### Concomitant immunomodulator use

Concomitant immunomodulator therapy during the SC infliximab phase was used in 30/58 patients (51.7%) overall: 20/25 (80.0%) in new starters and 10/33 (30.3%) in switchers; azathioprine was the immunomodulator in all cases. Post-treatment trough concentrations did not differ meaningfully by immunomodulator exposure: post-induction median trough in new starters was 17.5 µg/mL with concomitant immunomodulator and 16.0 µg/mL without; post-switch median trough in switchers was 34.5 µg/mL with concomitant immunomodulator and 36.5 µg/mL without. Anti-drug antibodies developed in 2/58 (3.4%) patients, both of whom were receiving concomitant azathioprine at the time of detection.

### BMI and SC infliximab levels

**Figure 4:**
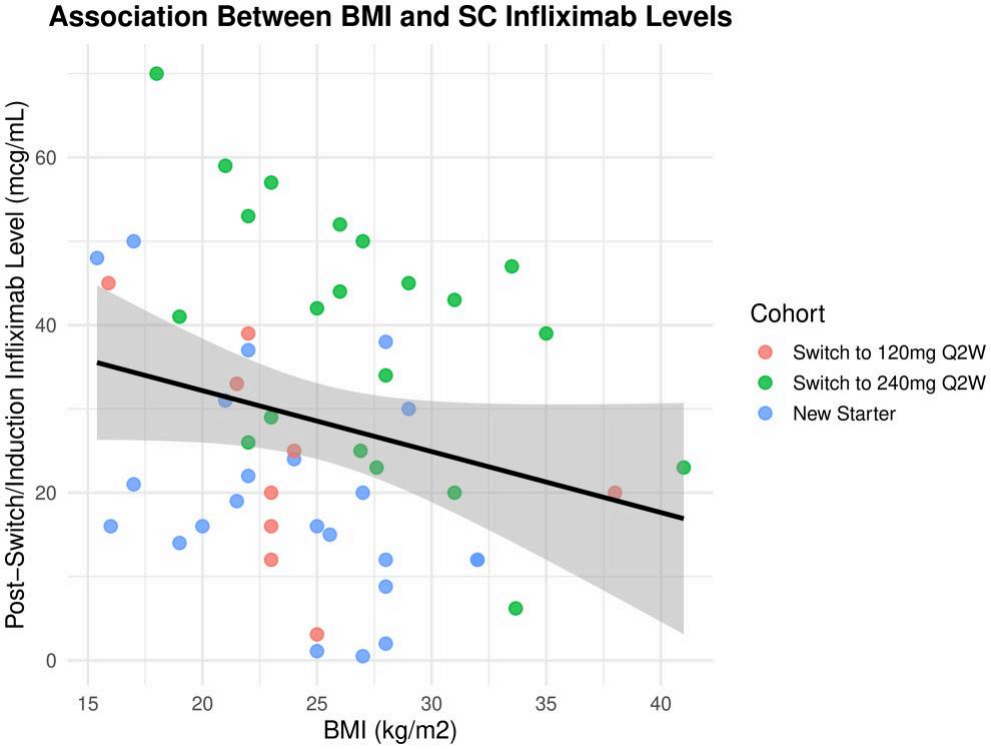
Correlation between body mass index (BMI) and infliximab trough levels. Scatter plot showing the relationship between BMI (kg/m^2^) and post-treatment infliximab trough levels (µg/mL) across all patients. Each point represents an individual patient’s trough level and BMI. A linear regression line with 95% confidence interval is overlaid. Pearson correlation: *r* = −0.24, *P* = .082.

An exploratory analysis of the relationship between BMI and SC infliximab levels was performed (Figure 4). No statistically significant correlation was observed between BMI and post-induction or post-switch SC infliximab trough levels across the entire cohort (Pearson’s *r* = −0.24; *P* = .082). Stratification by BMI category showed no significant differences in median trough levels: normal weight (BMI below 25 kg/m^2^; *n* = 25) median trough 29.0 (20.0, 45.0) mcg/mL; overweight (BMI 25-30 kg/m^2^; *n* = 19) median trough 23.0 (10.4, 40.0) mcg/mL; obese (BMI 30 kg/m^2^ or above; *n* = 9) median trough 20.0 (12.0, 39.0) mcg/mL (Kruskal−Wallis *P* = .194).

### Drug persistence

**Figure 5:**
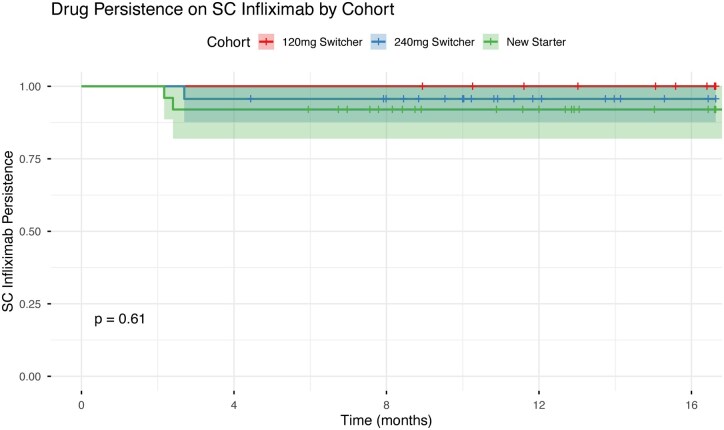
Kaplan–Meier curves for drug persistence by cohort. Kaplan–Meier estimates of drug persistence (time to treatment discontinuation) over 18 months for the 3 patient groups. No statistically significant difference was observed between groups tick marks indicate censored observations. Numbers at risk are shown below the *x*-axis.

Kaplan−Meier analysis of SC infliximab drug persistence is shown in Figure 5. Drug persistence was high with over 92% at 12 months across all cohorts. No statistically significant difference in drug persistence was observed between the 3 groups (log-rank *P* = .61). Among switchers, 3 patients reverted to IV infliximab (2 due to patient preference and 1 requiring re-induction following a patient-initiated drug holiday), while a further 4 permanently discontinued SC infliximab (3 due to treatment failure as detailed in the Pharmacokinetic Outcomes section, and 1 due to patient decision). No new starters required reversion to IV infliximab; however, 3 permanently discontinued SC infliximab due to patient-led cessation, immunogenicity in the context of prior infliximab exposure (as detailed in the Pharmacokinetic Outcomes section), and paradoxical psoriasis. The latter represented the only treatment-emergent adverse event leading to discontinuation identified in this cohort; no other safety events were recorded during the study period.

## Discussion

This is the first prospective, real-world study from the Middle East to evaluate both switching and de novo strategies for SC infliximab in IBD. Our findings demonstrate that both approaches are effective, leading to robust drug exposure, high rates of clinical remission, and durable treatment persistence, thereby addressing a significant geographic gap in the current literature.

A key finding is the substantial increase in infliximab trough concentrations following the IV-to-SC switch, consistent with data from pivotal trials and other large real-world cohorts. The increase was particularly pronounced for patients on intensified IV regimens who were switched to the 240 mg SC dose, confirming the appropriateness of this stratified dosing strategy. The REMSWITCH study similarly found that switching patients from highly intensified IV regimens (eg, 10 mg/kg every 4 weeks) to the standard 120 mg SC dose was associated with a high relapse rate, underscoring the need for a higher SC dose in this population.^7^ In the 120 mg switcher group, median trough levels rose from 9.5 to 20.0 mcg/mL post-switch, exceeding the TDM threshold of 12-13 mcg/mL associated with clinical remission in a prospective TDM-guided cohort.^9^ The robust trough levels achieved in our 240 mg group (median 42.0 µg/mL) also highlight a potential opportunity for TDM-guided dose de-intensification to avoid supra-therapeutic concentrations, which has both safety and cost implications. As detailed in the Results, a subset of switchers underwent TDM-guided de-intensification on this basis, illustrating the practical utility of bidirectional dosing in this setting. A target trough level of 20 mcg/mL or above has been proposed for achieving deeper outcomes including endoscopic and histologic remission, reinforcing the importance of TDM-guided management across all SC dosing groups.^9^

Our study provides novel real-world data on patients newly initiated on SC infliximab after standard IV induction. The median post-induction level of 16.0 µg/mL is consistent with levels reported in the LIBERTY trials and is consistent with trough concentrations associated with remission in published cohorts, with 88% of new starters achieving clinical remission at this timepoint.^4^ However, 28% of new starters required dose intensification from 120 to 240 mg Q2W during follow-up, suggesting that a flat starting dose of 120 mg is insufficient for a meaningful minority. This reinforces the utility of early post-induction TDM to identify patients who may benefit from proactive dose escalation, particularly those with more complex disease phenotypes or high inflammatory burden. While our median level lies within the range associated with remission, it may not be sufficient to achieve these more stringent targets associated with deeper outcomes in all patients, particularly those with perianal CD.

Our cohort included 23 patients with perianal CD across both groups, providing useful real-world data for this challenging phenotype. In new starters, 30% achieved perianal healing and a further 50% showed improvement following SC infliximab induction. In the switcher cohort, 77% of patients with perianal disease had achieved or went on to achieve perianal healing following the switch, with no patients experiencing perianal deterioration. Notably, maintenance of clinical remission was 100% in perianal switchers, compared to 85.7% in those without perianal disease. Trough levels were comparable between perianal and non-perianal patients in the switcher group, suggesting that the higher absolute concentrations achieved with SC infliximab (particularly at the 240 mg dose) may be sufficient to maintain adequate mucosal and fistular drug exposure in this population. These findings add to the growing evidence that SC infliximab is effective for perianal CD and support TDM-guided dose optimization in this phenotype.

Two patients developed neutralizing anti-infliximab antibodies despite combination therapy with azathioprine. Poor treatment adherence was the likely driver in 1 case, consistent with the established role of subtherapeutic drug levels in immunogenicity. In the second, pre-formed antibodies from a prior infliximab course were identified on re-exposure, indicating that antibody formation was not attributable to the SC formulation. Both cases highlight the importance of adherence monitoring and TDM, particularly in patients with prior biologic exposure.

The overall rate of concomitant immunomodulator use in our cohort (30/58, 51.7%) was substantially higher than the approximately 25.6% reported in REMSWITCH,^7^ reflecting both the high proportion of biologic-naïve new starters co-induced with azathioprine and the relatively early position of SC infliximab in the treatment sequence. However, within-cohort post-treatment trough concentrations did not differ meaningfully by immunomodulator exposure (new starters 17.5 versus 16.0 µg/mL; switchers 34.5 versus 36.5 µg/mL), and both observed cases of anti-drug antibody formation occurred in immunomodulator-exposed patients with subtherapeutic troughs and prior adherence concerns. We therefore do not interpret the favorable trough and immunogenicity profile as being principally driven by higher immunomodulator use; the high absolute SC infliximab concentrations achieved with maintenance dosing, particularly at 240 mg Q2W, are likely to be the dominant pharmacokinetic determinant. This interpretation is supported by the MINIMIZE randomized controlled trial, which evaluated withdrawal of thiopurines at the time of IV-to-SC infliximab switch and found that withdrawal did not increase immunogenicity, further suggesting that sustained drug exposure rather than concomitant immunomodulation is the principal contributor to the low immunogenicity observed in our cohort.^10^ A population-level contribution of higher immunomodulator coverage cannot be excluded, and confirmation in larger cohorts is warranted.

Our exploratory analysis revealed a near-significant inverse correlation between BMI and SC infliximab trough levels (*P* = .082), and a stepwise decrease in median trough levels across BMI categories was observed: 29.0 (20.0, 45.0) mcg/mL in normal weight patients, 23.0 (10.4, 40.0) mcg/mL in overweight patients, and 20.0 (12.0, 39.0) mcg/mL in obese patients, though this did not reach statistical significance. The consistent directional trend across all 3 categories suggests a clinically plausible BMI effect that the study was likely underpowered to confirm, given the small number of obese patients (*n* = 9). Notably, even in this group, median trough levels remained within the range associated with remission in published cohorts, which is reassuring. These findings align with a post-hoc analysis of the REMSWITCH study that found SC infliximab switching to be safe and effective in patients with obesity,^11^ but the trend observed here warrants prospective evaluation in larger cohorts with adequate power to characterize the relationship between body composition and SC infliximab pharmacokinetics.

Treatment persistence remained high (≥92%) during a median follow-up period of 10.1 months across all cohorts with no statistically significant difference between groups. These rates compare favorably with large prospective registries: the PEREM cohort reported 95.4% 1-year persistence after IV-to-SC switching,^8^ and an individual patient data meta-analysis across 3 European cohorts reported 94.5% 6-month persistence.^12^ The lower 12-month persistence in the 240 mg switcher group likely reflects this group’s more complex prior treatment history, including prior dose intensification, rather than any intrinsic limitation of the SC formulation. The convenience of at-home self-administration is likely a contributing factor to sustained adherence across all groups.

The main strength of our study is its prospective design, extended follow-up and novelty in providing real-world data from a middle eastern IBD population. By including 2 distinct and clinically relevant cohorts with dose stratification in switchers, we offer a comprehensive view of SC infliximab in routine practice. However, several limitations must be acknowledged. The observational, single-center design and modest sample size (58 patients) limit generalizability and statistical power, particularly constraining inference within subgroups, including the UC and perianal CD populations; these subgroup findings should therefore be regarded as hypothesis-generating and require confirmation in larger, multicenter cohorts. We lacked a contemporaneous IV control group. Adverse event reporting was not a prespecified outcome and was captured through routine clinical documentation, which may underestimate true incidence. Clinical and endoscopic outcomes were not systematically collected as primary endpoints, restricting interpretation of disease activity data. Finally, the significant difference in disease duration between cohorts may represent potential confounders when comparing outcomes between groups. Reassessment of clinical scores, biomarkers and infliximab trough concentrations was not performed at strictly protocolized timepoints but rather at the next available routine clinic visit within a defined window; this variability is inherent to real-world practice and is reported transparently, but limits direct comparison of timepoint-specific outcomes between patients. Perianal disease outcomes were assessed primarily by clinical examination; pelvic MRI was performed selectively rather than systematically, so we did not apply a validated radiological index (such as the Van Assche score or MAGNIFI-CD), and the perianal findings should be interpreted accordingly.^13^^,^^14^

## Conclusion

In conclusion, this prospective real-world study of IBD patients from the Middle East demonstrates that SC infliximab is an effective maintenance strategy for both IV-to-SC switchers and patients newly initiated after IV induction. Both cohorts achieved high rates of clinical remission, robust therapeutic drug concentrations, and durable treatment persistence. Switching to SC infliximab produces a substantial and sustained increase in infliximab trough concentrations, with dose selection informed by prior IV treatment intensity. These findings provide valuable real-world evidence from an underrepresented region and support the clinical utility of SC infliximab across diverse IBD populations.

## Supplementary Material

otag067_Supplementary_Data

## Data Availability

The data that support the findings of this study are available from the corresponding author upon reasonable request.
