# Supplementary material for: Real-world outcomes of subcutaneous infliximab in a Middle Eastern inflammatory bowel disease cohort: a prospective study of switch and de novo treatment strategies
Source: Crohns Colitis 360. 2026 Jun 27;8(3):otag067. doi: 10.1093/crocol/otag067 (PMC13371750; doi:10.1093/crocol/otag067)
Supplement: otag067_Supplementary_Data [file otag067_supplementary_data.docx]

**Supplementary Table 1. Clinical Outcomes in New Starter Patients Subgrouped by Disease Subtype**

Post-Induction Clinical Remission and Response Rates in IBD New Starters (n = 25) Stratified by Crohn’s Disease vs Ulcerative Colitis

| **Characteristic** | **Crohn’s Disease (n = 18)** | **Ulcerative Colitis (n = 7)** | **Total (n = 25)** |
| --- | --- | --- | --- |
| **Post-Induction Clinical Outcomes (Week 12–26)** | **CD (HBI < 5)** | **UC (PMS ≤ 2)** | **Combined** |
| **Clinical remission** | 16 / 18 (88.9%) | 6 / 7 (85.7%) | 22 / 25 (88.0%) |
| **Clinical responseᵃ** | 15 / 18 (83.3%) | 7 / 7 (100%) | 22 / 25 (88.0%) |

**ᵃ** Clinical response defined as a reduction of ≥3 points in Harvey-Bradshaw Index (HBI) for Crohn’s disease or ≥2 points in Partial Mayo Score (PMS) for ulcerative colitis from baseline.

HBI = Harvey-Bradshaw Index; PMS = Partial Mayo Score; SC IFX = subcutaneous infliximab; Q2W = every 2 weeks; CD = Crohn’s disease; UC = ulcerative colitis.

Note: Post-induction timepoint is defined as week 12–26 following subcutaneous infliximab initiation.

Data availability: biomarkers were analysed on a complete-case basis without imputation. CRP was available in 25/25 new starters and 30/33 switchers at baseline, and 25/25 and 31/33 post-treatment; faecal calprotectin in 20/25 new starters and 25/33 switchers at baseline, and 21/25 and 25/33 post-treatment. Combined biochemical remission was assessed only in patients with paired marker data (new starters n = 21, switchers n = 25).
